# Digital horizons in non-communicable disease care: a bibliometric exploration of intervention impact and innovation

**DOI:** 10.3389/fdgth.2025.1528711

**Published:** 2025-06-05

**Authors:** Sudip Bhattacharya, Alok Singh, Akanksha Kashyap

**Affiliations:** ^1^ Department of Community and Family Medicine, All India Institute of Medical Sciences, Deoghar (AIIMS Deoghar), Deoghar, India; ^2^ Department of Community Medicine, Faculty of Medicine and Health Sciences, Shree Guru Gobind Singh Tricentenary University, Gurugram, India; ^3^ Department of Ayurvedic Medicine, Research Scholar-Mahatma Gandhi Kashi Vidyapith, Varanasi, India

**Keywords:** telemedicine, m-Health (Mobile health), chronic disease, non-communicable diseases, digital health, health information systems

## Abstract

**Introduction:**

Digital interventions show considerable promise in managing non-communicable diseases (NCDs) within primary healthcare.

**Aim:**

The aim of this study was to conduct a comprehensive bibliometric analysis of research on digital interventions for individuals living with NCDs.

**Methodology:**

This study explores digital interventions in NCDs through a bibliometric analysis from 2014 to 2024. Carefully designed search queries targeted primary and combined terms to cover a wide range of NCDs, including cancer, diabetes, and hypertension. SCOPUS searches yielded 9,572 English-language articles, refined by excluding non-relevant works and duplicates. Metadata, including authorship, keywords, and citations, was extracted for analysis. Using Biblioshiny and VosViewer, the study examined publication trends, telemedicine applications, and the knowledge framework of the field. Conceptual themes were identified through co-occurrence mapping, intellectual structures via co-citation networks, and social structures through collaboration patterns among authors, institutions, and countries.

**Results:**

The upward trend in research on digital interventions and NCDs accelerated significantly after 2018, peaking in 2021, followed by a slight decline. Medicine dominates this field, with considerable contributions from biochemistry, health professions, and engineering. The most prolific authors, primarily from the United States, United Kingdom, and Australia, have significantly shaped this research area. Institutional contributions are led by Harvard Medical School and other global leaders, reflecting strong inter-institutional collaborations. The United States and the United Kingdom are the most productive countries, with the Journal of Medical Internet Research standing out as the leading publication. Keyword analysis reveals a focus on telemedicine, COVID-19, tele-health, and digital health. Co-citation analyses identify key intellectual frameworks, while co-authorship and institutional collaborations highlight robust global networks. Emerging trends emphasize AI, digital health tools, and patient self-management, underscoring a transformative shift in addressing NCDs through technology-driven interventions. The findings highlight the need for patient-centered applications, improved implementation strategies, and strengthened collaborations, especially in underrepresented regions, to enhance the global impact of digital interventions for NCDs.

## 1 Introduction

NCDs, as defined by the World Health Organization (WHO), are chronic conditions resulting from genetic, physiological, behavioral, and environmental factors. They account for nearly 63% of global mortality, causing over 36 million deaths annually, with 80% occurring in low- and middle-income countries (LMICs) (1). Digital interventions, endorsed by the WHO, offer a promising approach to improving NCD management by enhancing accessibility, continuity, and efficiency in healthcare delivery. The 2018 World Health Assembly recognized their potential to strengthen health systems by improving service delivery, empowering patients and providers, and achieving universal health coverage (2). WHO classifies digital health interventions into four user groups, encompassing 28 categories and 87 subcategories, with applications spanning prevention, diagnosis, treatment, and long-term disease management (3). Primary healthcare plays a crucial role in addressing NCDs, offering accessible and coordinated care for conditions like hypertension, diabetes, and cardiovascular diseases. However, LMICs face significant challenges, including shortages in human resources, medicine, equipment, and infrastructure, which hinder optimal NCDs management (4). Digital interventions, such as tele-health and mobile health (m-Health) applications, have demonstrated effectiveness in bridging these gaps. For instance, the CONNECT trial in Australia utilized a web-based app linked to electronic health records (EHRs), leading to improved blood pressure and lipid control (5). Similarly, the TEXTME trial showed that text messaging interventions resulted in better blood pressure management, healthier diets, increased physical activity, and smoking cessation among heart disease patients (6). Other studies highlight the effectiveness of digital tools in addressing medication adherence and weight management, particularly in high-income countries (7). India, with a population exceeding 1.3 billion, faces a rising NCDs burden, with conditions like heart disease, stroke, and diabetes projected to cost the country $54 billion annually (8). The India State-Level Disease Burden Initiative (2017) reported a significant increase in NCDs prevalence and mortality between 1990 and 2016, with cancer alone contributing to 8.3% of deaths and 5% of total disability-adjusted life years (DALYs) (9). Many Indians lack access to healthcare due to geographical barriers, infrastructure limitations, and financial constraints, emphasizing the need for scalable digital health solutions (10). Despite growing adoption, digital interventions in India remain primarily hospital-centered, limiting their reach to the broader population. While systematic reviews have highlighted the use of m-Health interventions in strengthening India's healthcare system (11), research on patient-centered digital interventions for NCDs management remains limited. A systematic review found that tele-health interventions were as effective as traditional care in improving the quality of life for cancer patients (12). Similarly, internet-based interventions have shown positive outcomes for patients with type 2 diabetes, cardiovascular diseases, stroke, osteoarthritis, depression, and chronic obstructive pulmonary disease (COPD) (13–36).

Apart from systemic review, bibliometric analysis is particularly valuable when the goal is to gain a comprehensive, data-driven understanding of a research field, its structure, key contributors, and emerging trends—something that systematic reviews and meta-analyses cannot capture as effectively. This approach provides a holistic view of the academic landscape, which can inform future research directions and policy decisions.

This study intended to address existing gaps by providing a comprehensive bibliometric analysis of digital interventions for NCDs, focusing on global research trends, key contributors, and emerging themes. While prior studies will have examined specific interventions in high-income countries, there will still be limited understanding of how these digital tools are studied and implemented in LMICs, where the burden of NCDs will remain highest. By analyzing publication patterns, collaboration networks, and thematic shifts, this study will offer a broader perspective on how digital health research evolves, particularly in resource-limited settings.

Furthermore, it will highlight underexplored areas, such as patient-centred digital interventions and the role of technology in primary healthcare, addressing the ongoing scarcity of evidence on their real-world impact. The novel insights from this analysis will include the identification of emerging research trends such as artificial intelligence, telehealth expansion, and mobile health applications for the self-management of NCDs. By mapping key research clusters and citation networks, this study will uncover gaps in digital health adoption, particularly in LMICs, where infrastructure and accessibility challenges will persist. Additionally, the findings will shed light on disparities in research funding and publication output between high-income and lower-income regions, emphasizing the need for targeted policy interventions. The study will also provide a roadmap for future research, highlighting areas where digital interventions could be optimized for greater impact, such as personalized healthcare, remote monitoring, and integration with existing health systems.

## 2 Aim

The aim of this study was to conduct a comprehensive bibliometric analysis of research on digital interventions for individuals living with NCDs.

## 3 Objectives

Our study objectives were to**-**

1. Analyze the growth and publication trends in research on digital interventions for managing NCDs over time.
2. Identify the most influential authors, institutions, and countries contributing to this research area.
3. Examine the collaborative networks among researchers and institutions in the field of digital interventions for NCDs management.
4. Determine the most frequently cited articles, journals, and keywords to reveal core topics and themes.
5. Explore research gaps and emerging trends to provide insights for future studies and potential improvements in digital interventions for NCDs.

## 4 Methodology

Searching queries were pinpointed and organised in the preliminary planning stage, as illustrated in Table 1. These queries were divided into two categories.

1. Primary search terms.
2. Combinations of primary search terms with NCDs-related items focused on digital Intervention in this field.

**Table 1:** List of keywords used.

TITLE-ABS-KEY(“digital interventions”) OR TITLE-ABS-KEY(“tele health”) OR TITLE-ABS-KEY(“e health”) OR TITLE-ABS-KEY(“e prescription”) OR TITLE-ABS-KEY(“e medicine”) OR TITLE-ABS-KEY(“e mental health”) OR TITLE-ABS-KEY(“tele surgery”) OR TITLE-ABS-KEY(“tele robotics”)) OR ((TITLE-ABS-KEY(“ncd”) OR TITLE-ABS-KEY(“cancer”) OR TITLE-ABS-KEY(“diabetes mellitus”) OR TITLE-ABS-KEY(“copd”) OR TITLE-ABS-KEY(“asthma”) OR TITLE-ABS-KEY(“sickle cell disease”) OR TITLE-ABS-KEY(“chronic kidney disease”) OR TITLE-ABS-KEY(“hypertension”) OR TITLE-ABS-KEY(“cerebral stroke”) AND TITLE-ABS-KEY(“digital health”) OR TITLE-ABS-KEY(“telemedicine”) AND PUBYEAR > 2013 AND PUBYEAR < 2025 AND [LIMIT-TO (SRCTYPE, “j”)] AND [LIMIT-TO (PUBSTAGE, “final”)] AND [LIMIT-TO (DOCTYPE, “ar”) OR LIMIT-TO (DOCTYPE, “re”)] AND (LIMIT-TO (LANGUAGE, “English”)))

The primary search terms encompassed “digital interventions”, and the secondary search terms included combinations related to NCDs. A subset of crucial search queries and their combinations were chosen based on their relevance to digital interventions and NCDs research. (Table- We broadened our literature review to encompass combinations of key search terms related to the use of digital interventions, focusing on NCDs. By including criteria for digital Interventions with terms like “NCDs, cancer, diabetes mellitus, COPD, asthma, sickle cell disease, chronic kidney disease, hypertension, and cerebral stroke”, our goal was to investigate how digital interventions are applied in these fields. This data is crucial for understanding the roles of digital intervention in NCDs. Moreover, specific research questions, outlined in Table 2, were formulated to provide a comprehensive overview of the knowledge structure and the bibliometric and statistical methods used to evaluate digital intervention research in NCDs from 2000 to 2024.

**Table 2:** Mapping of research questions with knowledge structure, bibliometric and statistical techniques.

Sl No	Research Questions	Knowledge structure covered	Bibliometric techniques
1.	What are the publishing trends of the research publication in digital interventions and NCDs research?	Intellectual structure	Annual Scientific Production.
2.	Who are the most contributing authors, journals, organisations, funding agencies and countries and cited papers in digital interventions and NCDs?	Intellectual structure	Three field plots, Most relevant Authors, Organisations, journal, funding agencies, Co-citation of author, journal and references
3.	What are the publication patterns and most frequently used keywords of the articles published in digital interventionsand NCDsresearch?	Conceptual structure	Network Analysis, Thematic mapping, and Thematic evolution and trending topics.
4.	What are the collaboration networks in digital interventions and NCDs research?	Social structure	Authors collaboration network, Institution collaboration network, and Country Collaboration network
5.	What are the thematic trends of the Application of digital interventions and NCDs research?	Conceptual structure	Thematic mapping, Thematic Evolution.
6.	What are the main open areas of challenges and the corresponding solutions for future research work in digital interventions and NCDs.	Conceptual structure	Thematic mapping, Thematic evolution, and Factorial analysis

### 4.1 Data collection

During the data collection phase, we systematically searched academic articles in the SCOPUS core collection from January 1, 2014, to Sept 30, 2024, focusing on digital intervention and NCDs research. Scopus was chosen for this bibliometric analysis due to its extensive coverage of peer-reviewed literature, interdisciplinary scope, and robust citation tracking. It provides a reliable dataset for evaluating publication trends, co-authorship networks, and thematic evolution. Additionally, Scopus offers built-in bibliometric analysis tools, making it a preferred choice for systematic literature reviews and trend assessments in academic research (36). The keywords employed for data retrieval are listed in Table 1. Additionally, English-language research articles and review papers were included in the study. This search yielded 9,572 academic publications from SCOPUS for analysis.

### 4.2 Data refinement

**Figure 1:**
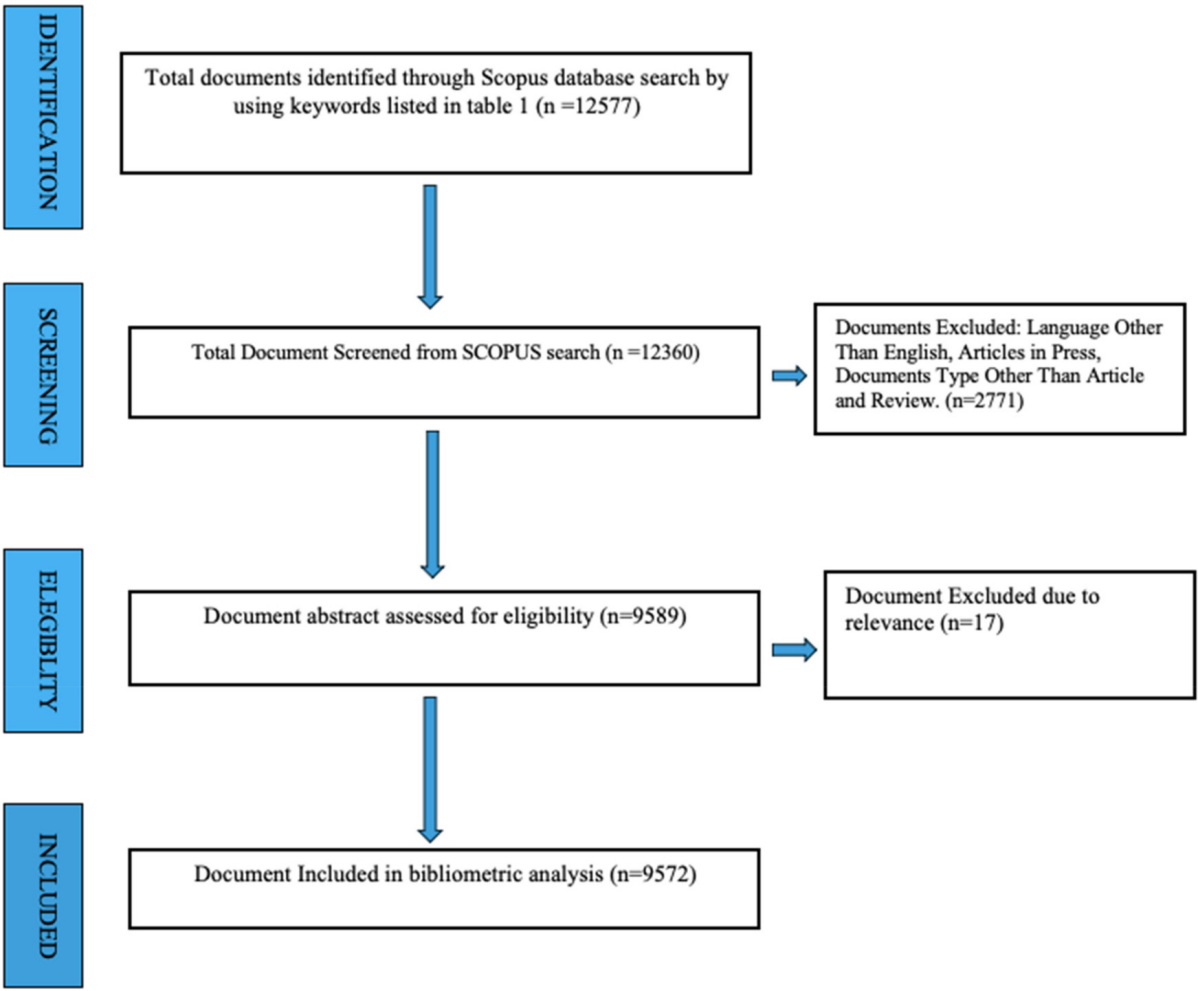
Results of keywords search as per PRISMA guidelines.

During the data refinement stage, we filtered the publications obtained from Scopus using specific exclusion criteria. We excluded books, editorials, letters, conference papers, and non-english academic works from our systematic bibliometric review. After this initial filtering, we removed duplicates and non-relevant articles such as articles not related to digital interventions, unrelated to NCDs, Research on general digital health tools that do not specifically target NCDs management, Non-primary studies like Opinion papers, editorials, commentaries, or letters that do not present original research or systematic reviews, from the remaining list of 9,589 publications, resulting in a final total of 9,572 articles (Figure 1).

### 4.3 Data extraction

We extracted metadata from Scopus in the form of a CSV bibliographic information file. The exported data included: (a) authors/editors, (b) full names of authors, (c) titles, (d) sources, (e) authors' keywords, (f) keywords plus, (g) abstracts, (h) authors affiliations, (i) corresponding authors affiliations, (j) cited references, (k) total citations, (l) highly cited papers, (m) usage counts, (n) publication years, (o) DOIs, (p) subject categories, (q) author identifiers, (r) languages, and (s) funding agencies.

### 4.4 Bibliometric analysis

Bibliometric analysis serves as an objective method for researchers to catalogue, access, and evaluate extensive collections of publications, offering a detailed overview of recent trends in scientific literature within a specific field or research area.

In this research, we conduct a bibliometric analysis of publications concerning the use of digital interventions research in NCDs from 2014 to 2024, addressing the six primary questions presented in Table 2. Different software tools and packages were utilized to analyse the retrieved data. **Biblioshiny (R package for Bibliometrics)**—Used for conducting bibliometric analysis and thematic mapping, making three field plots, Bradford law, and keyword analysis. **VOS viewer**—Employed to visualise co-authorship networks, keyword co-occurrence, and co-citation analysis to identify research clusters. **Microsoft Excel**—Used for data cleaning, refining search results, and structuring extracted bibliographic records. These tools collectively facilitated a comprehensive assessment of the knowledge structure, research collaborations, and thematic evolution in medical data breach research. Furthermore, we aim to statistically investigate and evaluate the scientific knowledge structure through this bibliometric analysis. The fundamental knowledge framework of a research field comprises three components:

1. **Conceptual structure**: Central themes and trends in the literature of a specific research area.
2. **Intellectual structure**: The impact of an author's work within the scientific community.
3. **Social structure**: Interactions among authors, institutions, and countries.

Initially, the conceptual structure is examined statistically using thematic mapping and co-occurrence networks. Subsequently, the intellectual knowledge structure is evaluated through co-citation network analysis. Lastly, the social knowledge structure is scrutinised based on the collaboration network and collaboration world map. By analysing these conceptual, intellectual, and social structures, we aim to comprehend the knowledge framework of telemedicine applications in mental health over the past decades. This analysis will highlight current achievements and identify future challenges in implementing the use of digital interventions in NCDs.

## 5 Results

### 5.1 Annual publications and trends

**Figure 2:**
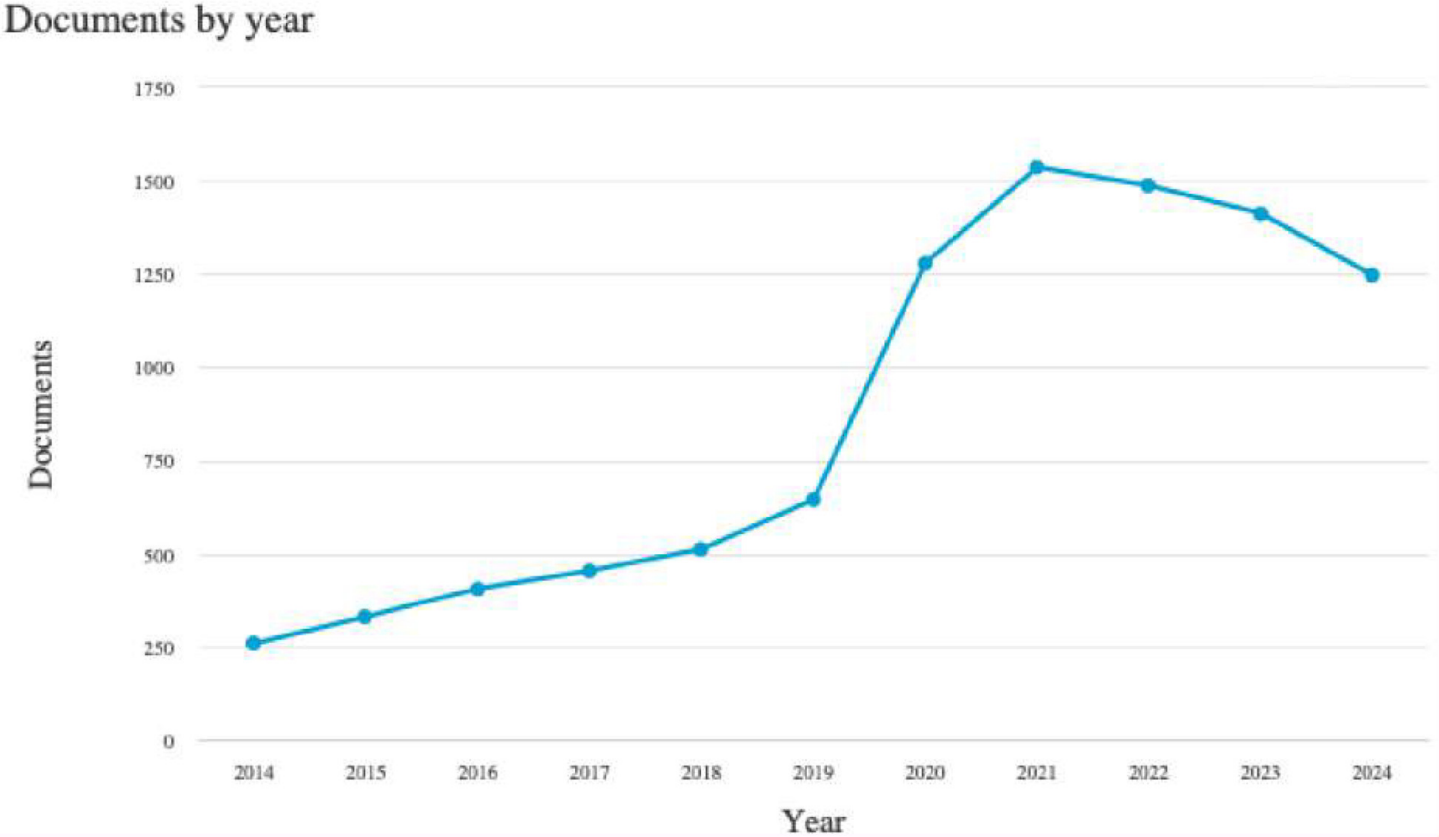
Annual scientific production (source; SCOPUS).

The data presented in Figure 2 illustrates a general upward trend in digital interventions and NCDs since 2021. Until 2018, annual publication numbers remained between 258 and 513. However, since 2018, a consistent year-on-year increase in publications was observed, with the year 2019 indicating a year of exponential growth, indicating that this research area entered a phase of rapid development. The highest output occurred in 2021, with 1,536 publications, and then, in the year 2021–24, the graph showed a small decline in publication. This suggests that digital interventions and NCDs research have garnered significant attention from the researchers' community during this period.

### 5.2 Analysis of the subject area

**Figure 3:**
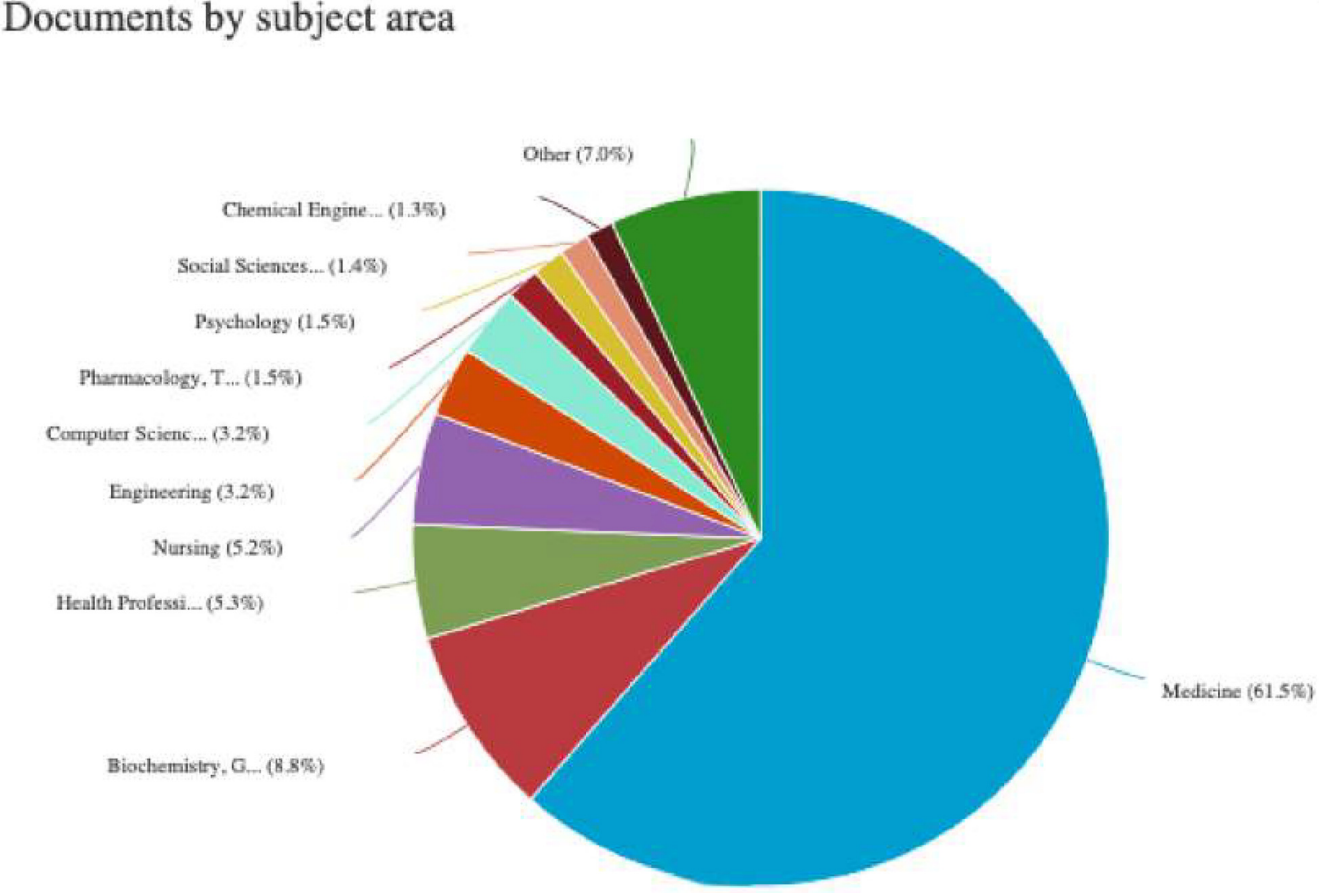
Documents by subject area (source: SCOPUS).

The pie chart in Figure 3 illustrates the distribution of research output related to digital interventions and NCDs across various subject areas. Most of the research is concentrated in Medicine, accounting for 61.5%, followed by Biochemistry, Genetics and Molecular Biology (8.8%). Other significant contributions come from Health Professions (5.3%), Nursing (5.2%), and Engineering (3.2%). Additionally, Computer Science represents (3.2%), Pharmacology, Toxicology and Pharmaceutics (1.5%), Psychology (1.5%), Social Sciences (1.4%), Chemical Engineering (1.3%), Others (7%). A detailed analysis confirms that Medicine is the predominant focus of this research.

### 5.3 Analysis of most relevant authors

A total of 51,858 authors participated in digital interventions and NCDs-related studies, with 10 authors contributing 20 or more papers each. Table 3 highlights these prolific authors, who collectively produced 248 publications, accounting for 2.5% of total submissions.

**Table 3:** Most productive authors.

Author Name	Country	Total Citations	Documents
Bosworth, H.B.	Duke Clinical Research Institute—Durham, United States	1,076	37
Yardley, L.	UK Health Security Agency—London, United Kingdom	1,142	34
McManus, R.J.	University of Oxford Medical Sciences Division—Oxford, United Kingdom	1,017	25
Pinnock, H.	The University of Edinburgh—Edinburgh, United Kingdom	1,114	24
Crowley, M.J.	Duke University School of Medicine—Durham, United States	269	23
Klonoff, D.C.	University of California, San Francisco—San Francisco, United States	230	22
Murray, E.	University College London—London, United Kingdom	876	21
Omboni, S.	Sechenov First Moscow State Medical University—Moscow, Russian Federation	783	21
Tarassenko, L.	University of Oxford—Oxford, United Kingdom (21)	1,263	21
Kario, K.	Jichi Medical University—Kawachi District, Japan		20
**Total Number of Documents**			**248**

Bosworth, H.B. from **D**uke Clinical Research Institute—Durham, United States, was the most productive, publishing 37 papers and 1,076 citations. He was followed by Yardley, L.from the UK Health Security Agency—London, United Kingdom (34 papers, 1,142 citations) and by McManus, R.J.fromthe University of Oxford Medical Sciences Division—Oxford, United Kingdom (25 papers, 1,017 citations). The research work by Dubey S et al. from the Institute of Post Graduate Medical Education & Research, Kolkata, India, titled “Psychosocial impact of COVID-19”, had the highest citation count, with 1,244 citations. The top 10 authors were primarily based in the United States, United Kingdom, Japan and the Russian Federation.

### 5.4 Analysis of the organisations

**Figure 4:**
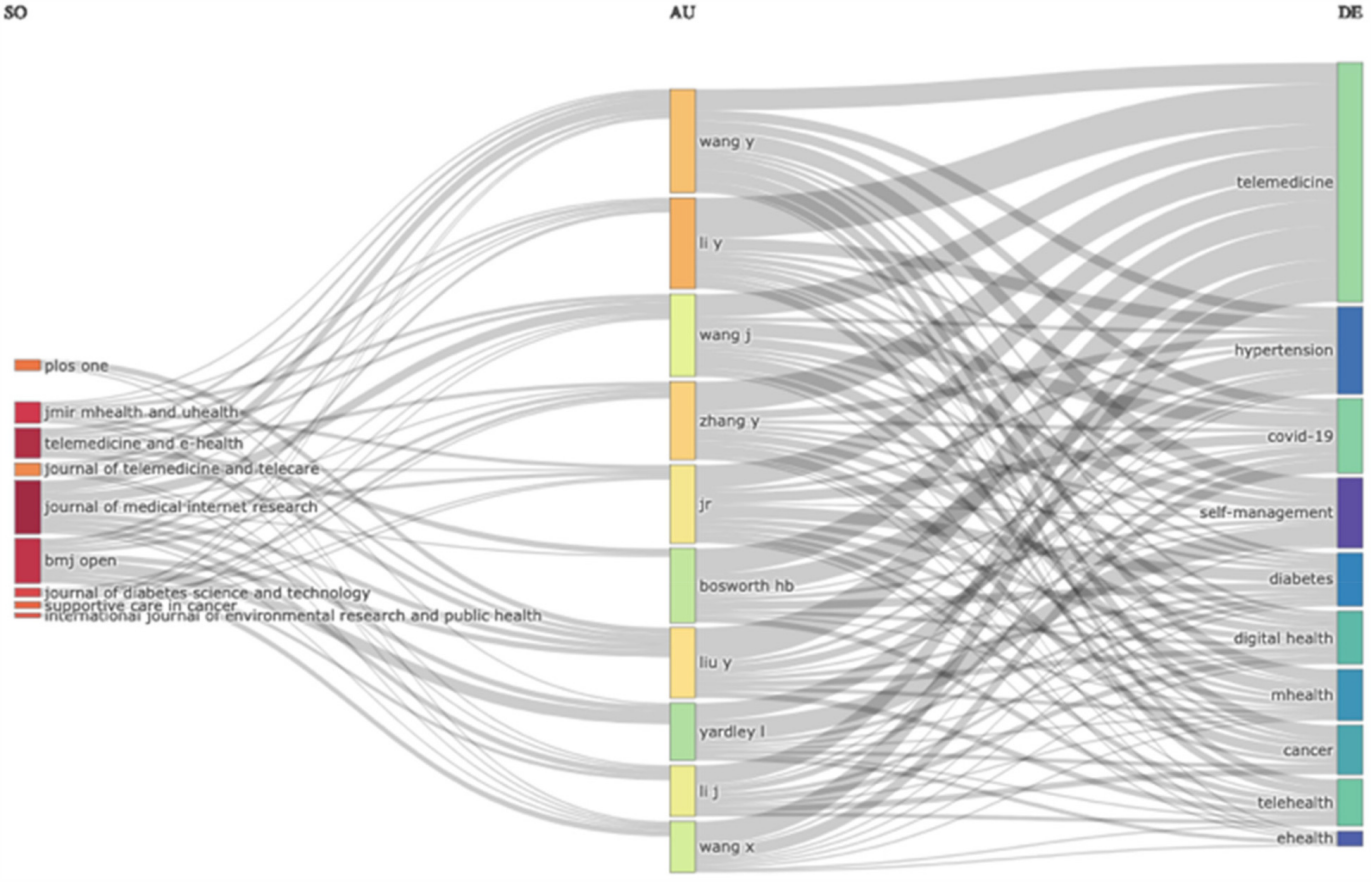
Three field plot.

The study analysed contributions from 37,779 distinct organisations, with the top 10 institutions contributing 1,594 publications. Harvard Medical School, USA, ranked highest with 263 papers, followed by the University of Toronto, Canada (193 papers) and the University of California, San Francisco, USA (177 papers). Other significant contributors included the University of Sydney, Australia,with 168 papers; Duke University School of Medicine, United States, with 146; and The University of Queensland, Australia,with 135 papers. The University of Melbourne, Australia,with 131 papers; Brigham and Women's Hospital, United States, with 130 papers;Imperial College London, UK, 126 papers; and Duke University,USA, 125 papers (Table 4). The top 10 organisations contributed 16.6% of total publications. Figure 4 uses a three-field plot diagram to illustrate the pattern of authors' publications in different related topics and journals.

**Table 4:** Top contributing organisations.

Affiliation	Country	Documents
Harvard Medical School	USA	263
University of Toronto	Canada	193
University of California, San Francisco	USA	177
The University of Sydney	Australia	168
Duke University School of Medicine	USA	146
The University of Queensland	Australia	135
University of Melbourne	Australia	131
Brigham and Women's Hospital	USA	130
Imperial College London	UK	126
Duke University	USA	125
**Total Number of Documents**		**1,594**

### 5.5 Analysis of country scientific production

Table 5 lists the ten leading countries involved in digital interventions and NCDs related to research. Table 5 provides data on the total number of published articles in this field by different countries. According to Table 5, only the USA and the UK have published over 1,000 papers on digital interventions and NCDs from 2004 to 2024. The USA is the leading country in terms of scientific productivity, with 3,884 publications, followed by the United Kingdom (*n* = 1,196) andAustralia (*n* = 751).

**Table 5:** Top contributing countries.

Country	Documents
United States	3,884
United Kingdom	1,196
Australia	751
Canada	608
Italy	602
Germany	503
China	469
Netherlands	444
India	417
Spain	412
**Total number of Documents**	**9,286**

### 5.6 Most preferred journal

A total of 2,214 academic journals have published research articles on *digital interventions* and NCDs. The Ten most active journals accounted for 1,711 out of 9,572 papers, contributing 17.8% of the total publications (Table 6).

**Table 6:** Most prolific journals.

Journals	Documents	Quartile	H index	Documents
Journal Of Medical Internet Research	429	Q1	197	429
Telemedicine And E Health	230	Q1	94	230
BMJ Open	197	Q1	160	197
Jmir M-health And U-health	153	Q1	96	153
Journal Of Diabetes Science And Technology	139	Q1	93	139
International Journal Of Environmental Research And Public Health	134	Q2	198	134
Supportive Care In Cancer	124	Q1	135	124
Plos One	117	Q1	435	117
Journal Of Telemedicine And Telecare	104	Q1	90	104
Diabetes Technology And Therapeutics	84	Q1	103	84
**Total number of Documents**	**1,711**			

The Journal Of Medical Internet Research led with 429 articles, followed by Telemedicine And E-Health (230), BMJ Open (197), Jmir M-health And U-health (153) and the Journal Of Diabetes Science And Technology (139). Other significant contributors were International Journal Of Environmental Research And Public Health (134), Supportive Care In Cancer (124), Plos One (117), Journal Of Telemedicine And Telecare (117), and Diabetes Technology And Therapeutics (84) (Table 6).

**Figure 5:**
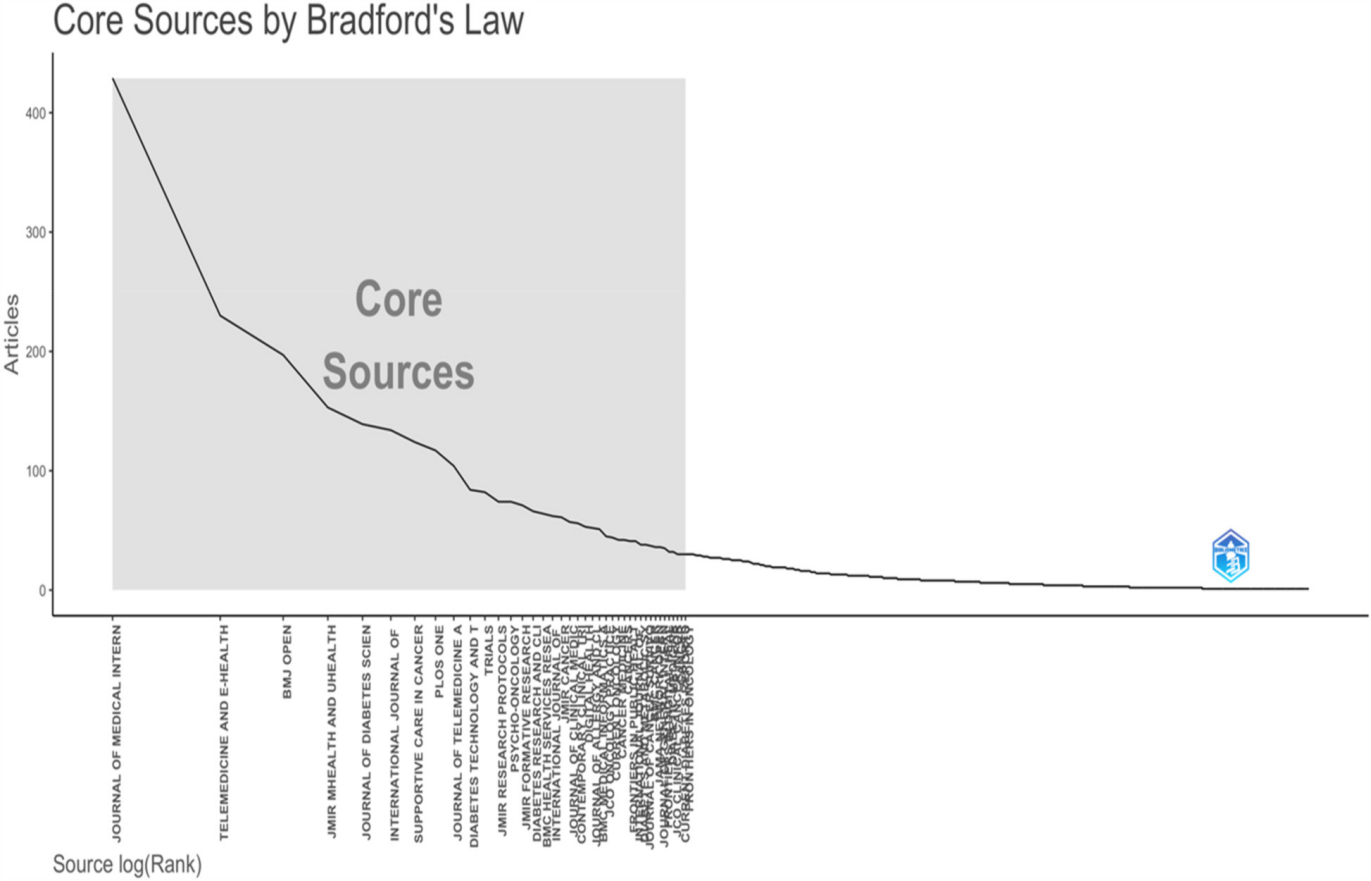
Bradford law.

Bradford's Law suggests that a small number of journals are central to a specific research field. As illustrated in Figure 5, the ten journals highlighted in Table 6 form this core group, accounting for approximately one-third of the entire collection's documents. Table 6 provides details about the country, quartile, and H index, as well as documents from the top ten periodicals, each contributing more than 84 articles to our bibliographic collection.

**Figure 6:**
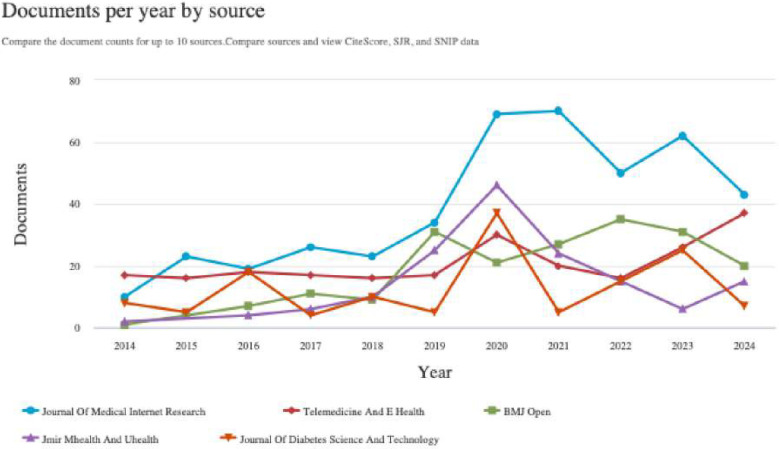
Documents per year by top journals (source: SCOPUS).

The Journal of Medical Internet Research has been publishing on *digital interventions* and NCDs since before 2014. It has been the leading publisher in the field since then, reaching its peak in 2020 and 2021. The Journal Telemedicine and E-Health is the second most contributing journal in this field in the year 2014 to 2018 and in the year 2024 (Figure 6).

### 5.7 Analysis of the highly cited research publications in *digital interventions*and NCDs

The ten most frequently cited research publications in the field of *digital interventions*and NCDs-related research, specifically within the analysed collection and published between 2014 and 2024, are listed in Table 7. For instance, Dubey S et al. from the Institute of Post Graduate Medical Education & Research, Kolkata, India, authored an article titled “Psychosocial impact of COVID-19”, which was published in the journal Diabetes and Metabolic Syndrome: Clinical Research and Reviews is the highest globally cited publication with 1,244 citations. The second most influential paper, with 990 total citations, is in the 2023 ESC Guidelines for the Management of acute coronary syndromes by Byrne RA.et al., published in the European Heart Journal in the year 2023. Article Machine Learning and Data Mining Methods in Diabetes Research by KavakiotisI also garnered 925 total citations. Lastly, Bakitas MA et al.'s publication, Early vs. delayed initiation of concurrent palliative oncology care: Patient outcomes in the ENABLE III randomized controlled trial, achieved 864 total citations, as depicted in Table 7.

**Table 7:** Highly cited research publications on digital interventions for NCDs.

Title	Year	Source title	Cited by	DOI
Psychosocial impact of COVID-19 (37)	2020	Diabetes and Metabolic Syndrome: Clinical Research and Reviews	1,244	10.1016/j.dsx.2020.05.035
2023 ESC Guidelines for the management of acute coronary syndromes (38).	2023	European Heart Journal	990	10.1093/eurheartj/ehad191
Machine Learning and Data Mining Methods in Diabetes Research (39).	2017	Computational and Structural Biotechnology Journal	925	10.1016/j.csbj.2016.12.005
Early vs. delayed initiation of concurrent palliative oncology care: Patient outcomes in the ENABLE III randomized controlled trial (40).	2015	Journal of Clinical Oncology	864	10.1200/JCO.2014.58.6362
Advances in paper-based point-of-care diagnostics (41).	2014	Biosensors and Bioelectronics	850	10.1016/j.bios.2013.10.075
Impact of mHealth chronic disease management on treatment adherence and patient outcomes: A systematic review (42).	2015	Journal of Medical Internet Research	792	10.2196/jmir.3951
Diabetic retinopathy: global prevalence, major risk factors, screening practices and public health challenges: a review (43).	2016	Clinical and Experimental Ophthalmology	704	10.1111/ceo.12696
The impact of mHealth interventions: Systematic review of systematic reviews (44).	2018	JMIR mHealth and uHealth	669	10.2196/mhealth.8873
Predictors of ehealth usage: Insights on the digital divide from the health information national trends survey 2012 (45).	2014	Journal of Medical Internet Research	667	10.2196/jmir.3117
Practical recommendations for the management of diabetes in patients with COVID-19 (46).	2020	The Lancet Diabetes and Endocrinology	640	10.1016/S2213-8587 (20)30152-2

### 5.8 Active funding agencies

Of the 9,572 articles reviewed, 566 were funded by the top 10 organisations. The National Institutes of Health in the United States was the largest contributor, backing 1,015 *digital interventions* and NCDs-related studies. Other major funders included the U.S. Department of Health and Human Services (*n* = 676), the National Cancer Institute (*n* = 415), National Institute of Diabetes and Digestive and Kidney Diseases (*n* = 224),National Center for Advancing Translational Sciences (*n* = 202), National Heart, Lung, and Blood Institute (*n* = 198), European Commission (*n* = 190) National Health and Medical Research Council (*n* = 147), National Institute for Health Research (*n* = 112), National Institute for Health and Care Research (*n* = 109). Six out of the top ten funders were from The United States, two from the United Kingdom and one from the European Union and Australia (Table 8).

**Table 8:** Top funding bodies.

Funding Bodies	Country	Documents
National Institutes of Health	United States	1,015
U.S. Department of Health and Human Services	United States	676
National Cancer Institute	United States	415
National Institute of Diabetes and Digestive and Kidney Diseases	United States	224
National Center for Advancing Translational Sciences	United States	202
National Heart, Lung, and Blood Institute	United States	198
European Commission	European Union	190
National Health and Medical Research Council	Australia	147
National Institute for Health Research	United Kingdom	112
National Institute for Health and Care Research	United Kingdom	109
**Total Funded Documents**		**566**

### 5.9 Conceptual knowledge structure analysis

#### 5.9.1 Analysis of the keywords

In this part of our study, we employed keyword and co-occurrence analyses to explore the latest research trends in *digital interventions* and NCDs. “Our objective is to pinpoint research gaps and predict future directions in this domain”.

**Figure 7:**
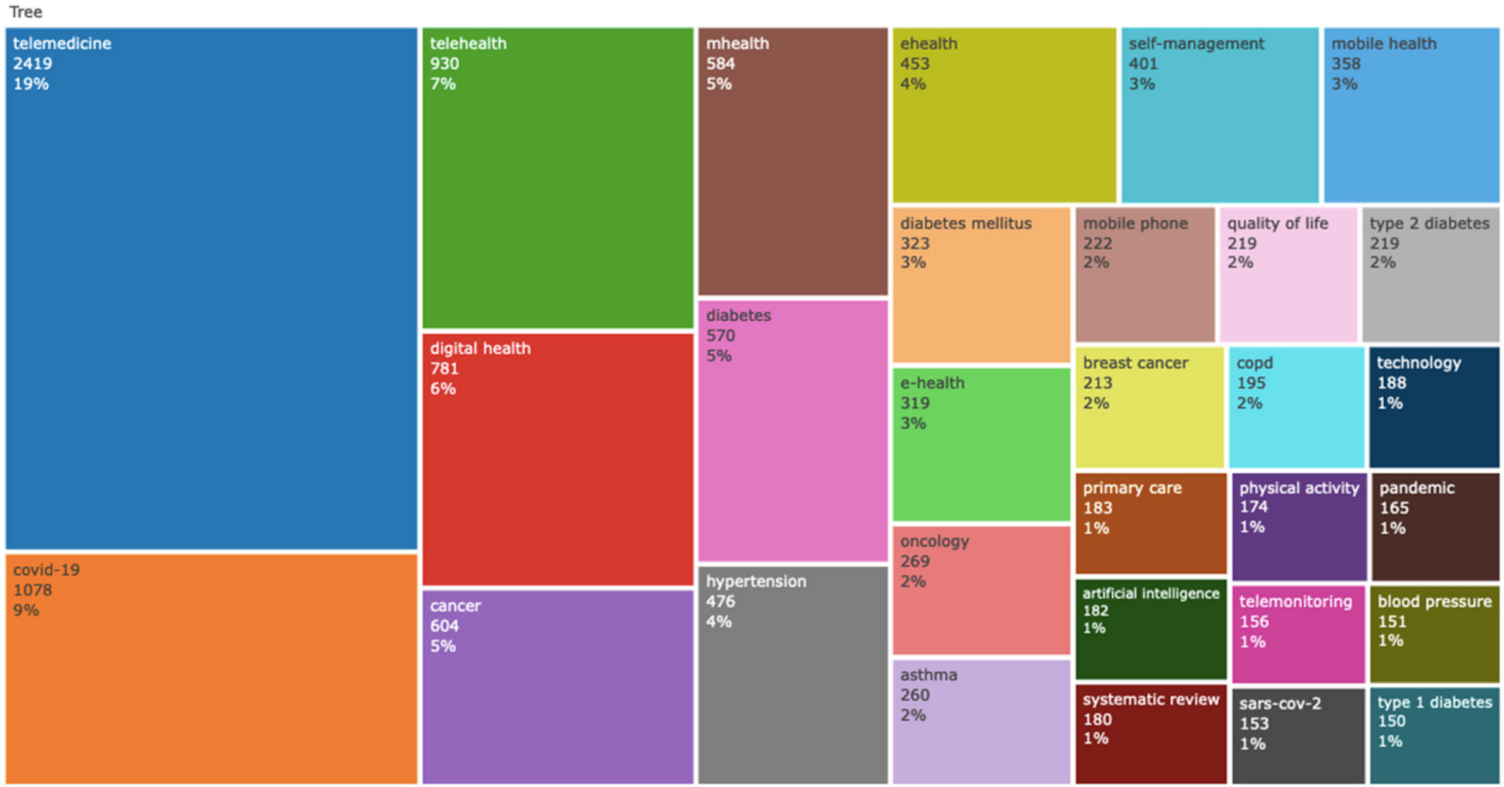
Tree map of authors keywords.

**Figure 8:**
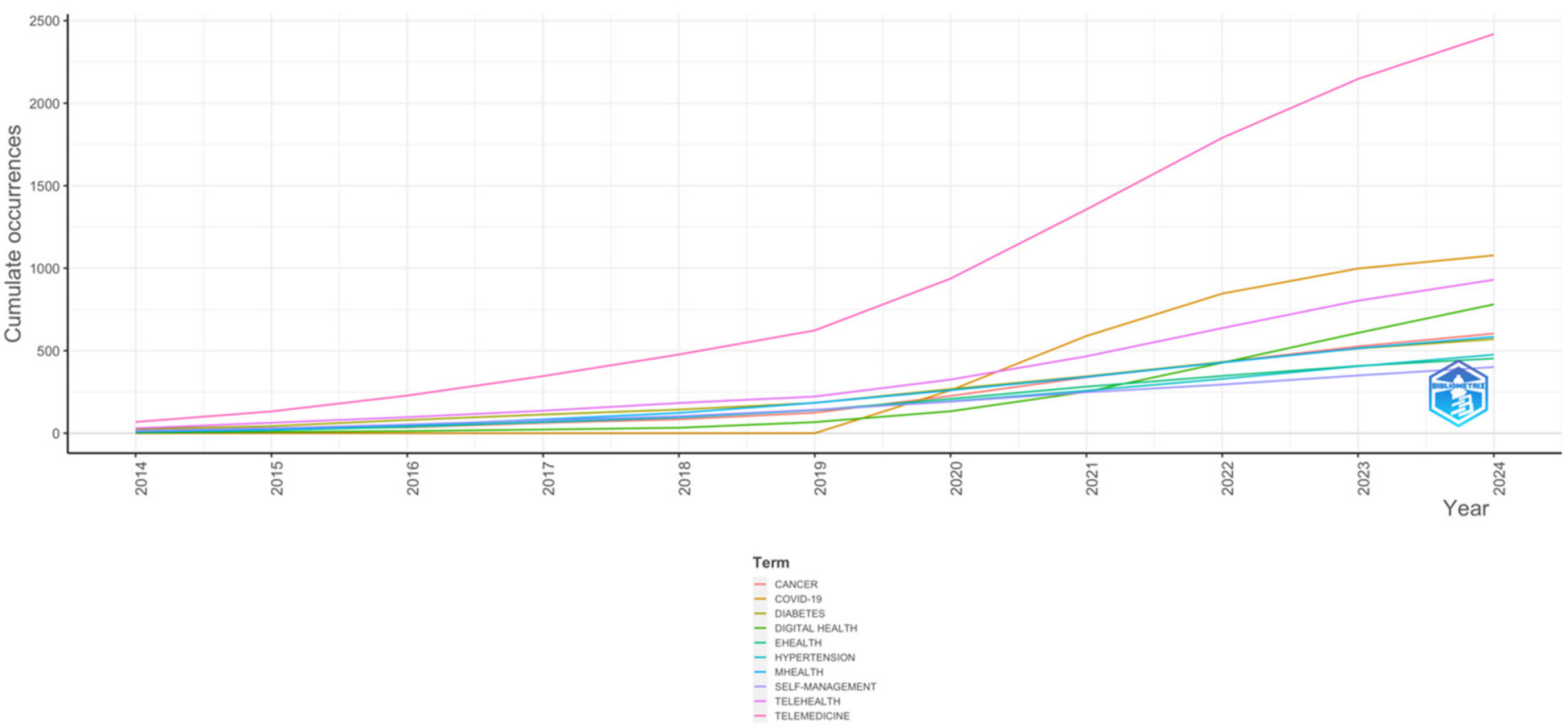
Word frequency over time.

The primary keywords are illustrated in Figure 7, with “TELEMEDICINE” (2,419 occurrences) being the most frequent, followed by “COVID-19” (1,078 occurrences), “TELEHEALTH” (930 occurrences), “DIGITAL HEALTH” (781 occurrences), and “CANCER” (604 occurrences) “MHEALTH” (584 occurrences), “DIABETES” (570 occurrences), “HYPERTENSION” (476 occurrences), E-HEALTH (453 occurrences), SELF-MANAGEMENT (401 occurrence). The relationship between digital interventions and NCDs research is also depicted using the cumulative occurrence graph in Figure 8. This graph shows a progressive increase in the annual occurrence of the author's keywords related to assistive devices, assistive technology and disability. Authors keywords such as TELEMEDICINE, COVID-19, TELEHEALTH, DIGITAL HEALTH, CANCER, MHEALTH, DIABETES, HYPERTENSION, EHEALTH, SELF-MANAGEMENT and these terms have exhibited more dynamic growth compared to others. The comparison between the author's main keyword cumulative occurrence since the year 2014and 2024 shows that the main author keywords have increased significantly, indicating trending or popular research topics in the field, growing interest or emerging trends in that topic. The graph highlights the shifts in research focus.

Furthermore, we analysed the co-occurrence of authors' keywords using Vos Viewer. The co-occurrence network helps understand the thematic areas of the research field and identify the most critical and current issues. It also provides insights into the evolution of these issues over time (47, 48).

**Figure 9:**
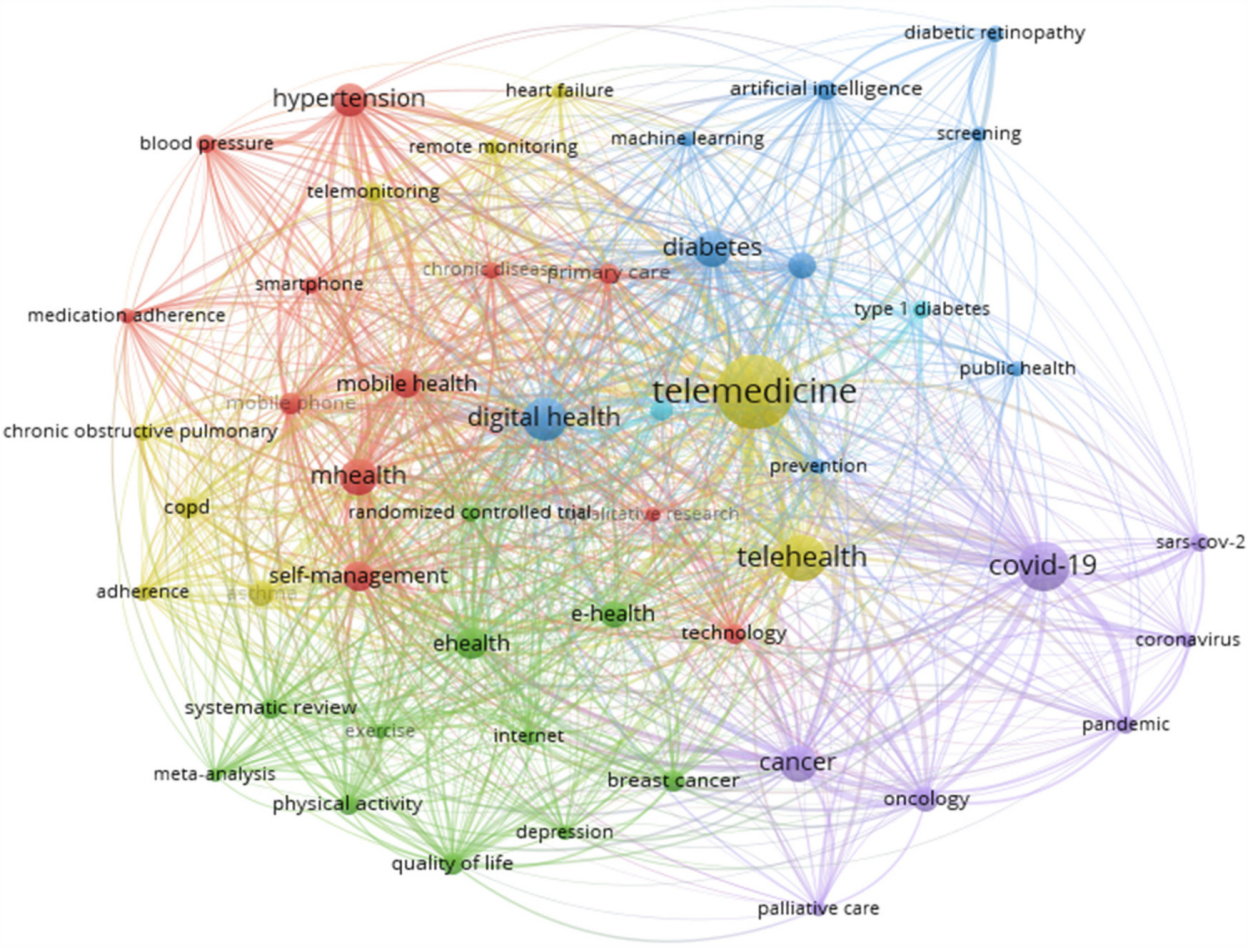
The network visualisation map of keywords Co-occurrence.

We extracted 50 out of 12,343 Authors’ Keywords with a frequency of 90 or more for co-occurrence analysis in Vos viewer to explore trends and hotspots in the field of research (Figure 9). The results of the co-occurrence network analysis are presented in Table 9. The visual representations classify keywords into five clusters, as shown in Table 9.

**Table 9:** Details of keywords in different clusters of network co-occurrence of authors’ keywords and key theme.

Cluster number/ no of Items/ Colour	Keywords of the clusters	Key Theme
Cluster 1 (12 items) **Red Colour**	blood pressure, chronic disease, hypertension, medication adherence, mhealth, mobile health, mobile phone, primary care, qualitative research, self-management, smartphone, technology	The theme indicated by this cluster centers on *mobile health (mHealth) and technology-driven self-management for chronic disease care*, focusing on areas like hypertension, medication adherence, and primary care support through mobile devices and digital interventions
Cluster 2 (11 items) green	breast cancer, depression, e-health, ehealth, exercise, Internet, meta-analysis, physical activity, quality of life, randomized controlled, systematic review	The cluster likely indicates a theme focused on *digital health interventions and lifestyle modifications* aimed at managing and improving quality of life for individuals with NCDs, particularly through *e-health tools, mental health support, and physical activity programs*.
Cluster 3 (9 items) Blue	artificial intelligence, diabetes, diabetes mellitus, diabetic retinopathy, digital health, machine learning, prevention, public health, screening	The theme of this cluster likely centers on “**Digital Health and AI-Driven Approaches in Diabetes Prevention and Screening”**, focusing on using artificial intelligence and machine learning in digital health interventions for public health and NCDs prevention, particularly in diabetes and its complications like diabetic retinopathy
Cluster 4 (9 items) Yellow	adherence, asthma, chronic obstructive pulmonary disease, copd, heart failure, remote monitoring, telehealth, telemedicine, telemonitoring	This cluster likely represents a theme focused on *remote healthcare management for chronic respiratory and cardiovascular conditions*, emphasizing adherence, telehealth technologies, and monitoring for conditions like asthma, COPD, and heart failure
Cluster 5 (7 items) Purple	cancer, coronavirus, covid-19, oncology, palliative care, pandemic, sars-cov-2	This cluster likely indicates a theme focused on the intersection of *digital interventions in managing and supporting NCDs* amid *pandemic-related challenges,* particularly in *cancer care, palliative care, and COVID-19-related adjustments*
Cluster 6 (2 items) Purple	type 1 diabetes, type 2 diabetes	The presence of keywords like “type 1 diabetes” and “type 2 diabetes” in a cluster likely indicates a theme focused on *diabetes management and intervention* within the broader context of digital interventions for noncommunicable diseases

#### 5.9.2 Thematic Map

**Figure 10:**
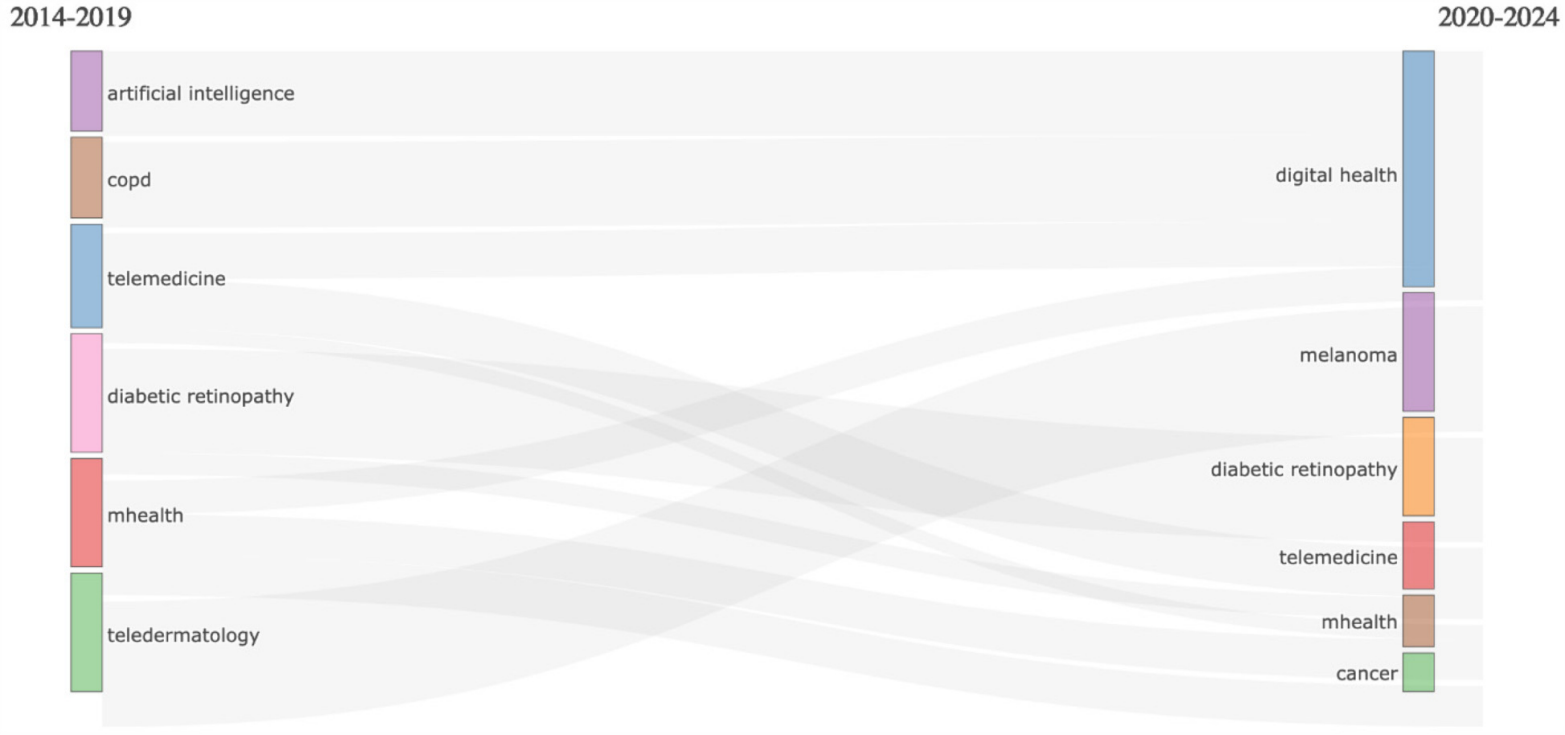
Sankey diagram.

**Figure 11:**
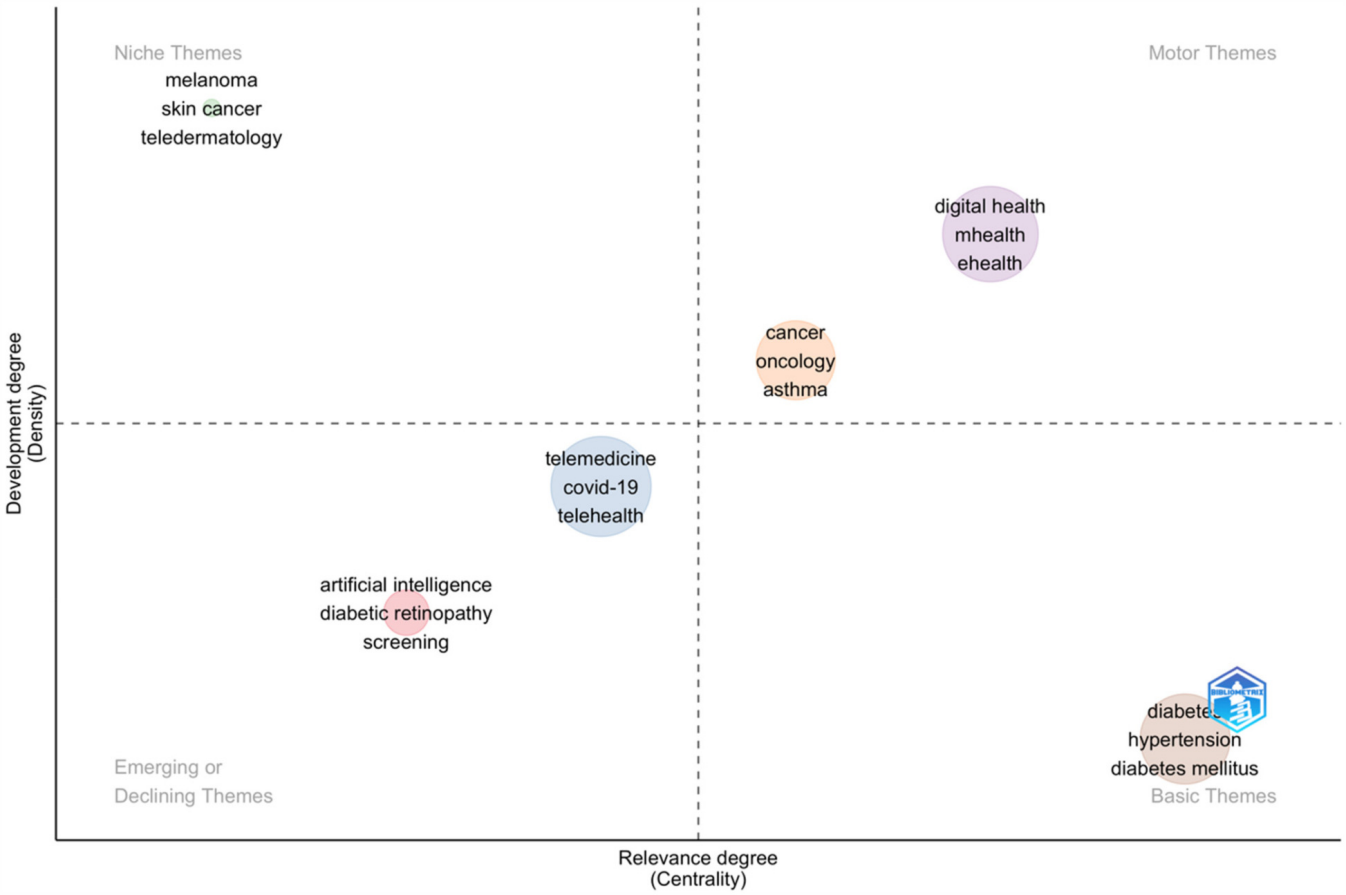
Thematic map.

The thematic development of keywords from 2014 to 2024 is examined using the keyword thematic map and Sankey diagram presented in Figure 10. Analysing the thematic map helps researchers to understand the current landscape of their field, identify key themes, and explore areas that need further investigation. The map divides these themes into four quadrants based on two axes. Centrality (*X*-axis) indicates the importance or relevance of the theme in the overall research field, and Density (*Y*-axis) indicates the theme's internal development, i.e., how well-developed and mature the theme is. The Upper-Right Quadrant (Motor Themes) is related to High Centrality and High Density. These themes are both important and well-developed. They play a significant role in structuring the research field and are actively developed, serving as “motor” themes (49). These include themes like digital health, m-health, e-health, oncology, cancer and asthma. Upper-Left Quadrant (Niche Themes) is related to Low Centrality and High Density. These themes are well-developed but not central to the overall research field. They often represent specialised or niche topics that are mature but not crucial to the broad field. It includes themes like melanoma, skin cancer and tale dermatology. The lower-Right Quadrant (Basic and Transversal Themes) is related to High Centrality and low Density. These themes are important but underdeveloped. They represent foundational or emerging areas that have relevance across various subfields but require further development. It includes themes like Diabetes, Hypertension and Diabetes Mellitus. The lower-Left Quadrant (Emerging or Declining Themes) is related to Low Centrality and low Density. These themes are neither well-developed nor important to the overall research field. They could represent either emerging themes that are yet to gain attention or declining themes that are losing relevance, and they include themes like Artificial Intelligence, Diabetic retinopathy and screening (Figure 11).

#### 5.9.3 Trending topics

**Figure 12:**
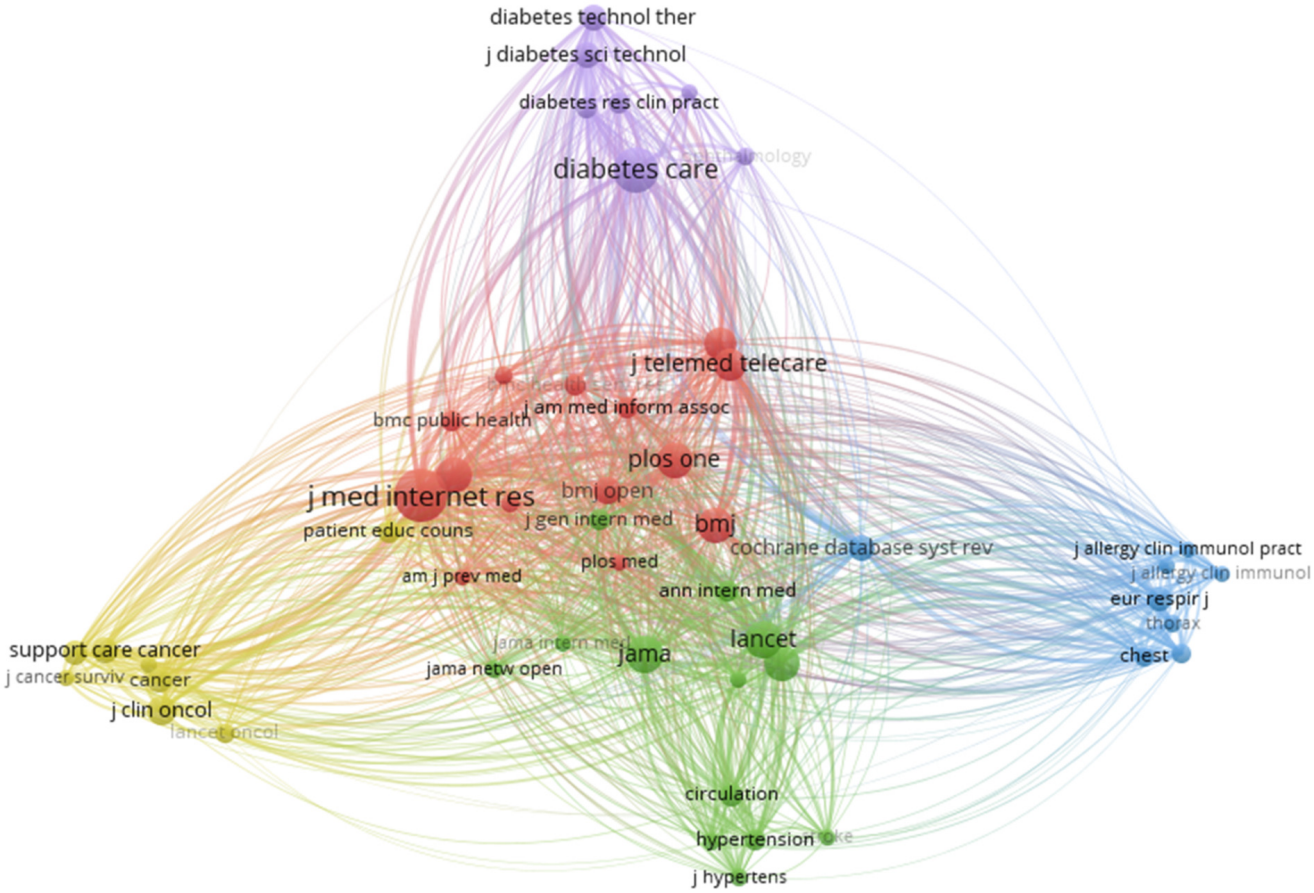
The network visualisation map of co-cited references.

We focused on two keywords appearing at least five times each year to analyse the trending topics. Since 1984,approximately 45 keywords have shown notable increases in frequency. Figure 12 illustrates the occurrence trends, highlighting keywords such as internet, self-care, telephone, artificial pancreas, complex intervention, self-management support, home health monitoring, behavioral health, care management and electronic mailgaining prominence in 2014–29.

This suggests that these research areas are currently receiving considerable attention and may reflect a strong focus on digital tools and strategies aimed at enabling self-management, enhancing patient engagement, and improving healthcare delivery for individuals with NCDs. But after 2019, themes like conversational agents, colorectal cancer screening, artificial intelligence, implementation science, digital health, oncology, telemedicine, COVID-19, e-health, and COPD have gained significant attention in the field indicates a comprehensive push towards using digital tools to enhance healthcare delivery, particularly for managing and preventing NCDs. This focus is likely a response to rising NCDs rates and the need for more efficient healthcare solutions. These themes are likely to remain focal points in future research on *digital interventions* and NCDs.

### 5.10 Analysis of Intellectual knowledge structure

#### 5.10.1 Analysis of co-cited authors

Co-cited authors network analysis refers to the examination of relationships between authors based on how often they are cited together in the same documents. This visualisation or map shows the relationships between authors based on their co-citation frequency. It helps identify groups or clusters of authors who are frequently referenced together, often indicating that they contribute to similar research areas or themes. In VOS viewer, these clusters of co-cited authors may represent specific research fields or topics. Authors within the same cluster are likely to have influenced each other or contributed to a shared body of knowledge. Co-cited author's network analysis can be used to identify leading scholars, influential research collaborations, or emerging trends in a particular academic discipline. Each author's publication is shown in a circle and denoted by the author's name. The colour of a publication shows the cluster to which the author's publication belongs. The size of each node in the graph represents the number of citations an author has received, with larger nodes corresponding to higher citation counts and greater influence (50). Of 51,851 authors, using 307 as a minimum number of co-citations, 50 met the threshold Wang.Yleads with 852 co-citations, followed by Michie. S, Altman. D.G,Li.Y and Li.Jwith **712**, **699**, **646** and **619** co-citations. The top 10 co-cited authors amassed over5609 co-citations, underscoring their significant impact on *digital Interventions* and NCDs.Co-authorship networks show strong institutional and national collaborations, with researchers from leading institutions like *Harvard Medical School, the University of Toronto,* and the *University of Oxford* frequently co-authoring publications.

#### 5.10.2 Analysis of co-cited journals

Co-cited journal network analysis examines relationships between journals based on how frequently they are co-cited together in other academic papers. Co-citation occurs when two journals are cited together by a third, meaning they are both referenced in the same document. The relationships between journals are visualised as a network, where journals are represented as nodes, and the strength of co-citations forms the edges connecting these nodes. Frequently, co-cited journals tend to form clusters, indicating that they share similar or related topics, research areas, or disciplines. The analysis helps identify core journals in a field and relationships between journals, revealing trends, disciplines, or interdisciplinary connections, as well as research fronts, and showing emerging fields of study. The larger the number of co-citations, the stronger the relationship between the journals, and this is reflected in the distance between nodes and the thickness of the connecting lines in the network visualisation (51). Figure 12 illustrates that the three core journals in the field are the **Journal of Medical Internet Research** (Q1, H index = 216),**Diabetes Care** (Q2, H index = 418), J**AMA-Journal of Medical Association** (Q1, H index = 768), **The Lancet** (Q1, H index = 895), **JMIR mHealth and uHealth**, (Q1, H index = 96).

#### 5.10.3 Analysis of co-cited references

Co-cited reference network analysis visualises a research field's intellectual structure by showing which references are frequently cited together, reflecting thematic relationships within the literature. In co-cited reference network analysis, nodes represent the references, and the links (edges) between them represent how frequently they are co-cited. A dense cluster of nodes in such a network suggests that the references in that cluster are often co-cited together, implying a close intellectual relationship or a thematic similarity. Groups of studies that are frequently cited together could reveal key themes, methodologies, or theories in a specific domain (52). Highly co-cited references often indicate foundational or highly influential works in a particular research area. Our analysis reveals that these ten articles primarily focus on four main research themes. (1) Methodological Approaches in digital Health Research, (2) digital Health Interventions and Their Application in NCDs, (3) Behavioral and Theoretical Foundations of digital Health (Table 10).

**Table 10:** Top 10 references in the field of digital intervention for NCDs research from 2014 to 2024.

Sl. No	Cited Article	Citations	Total link strength
1.	Statistical Power Analysis for the Behavioral Sciences	70	8
2.	Using thematic analysis in psychology	61	20
3.	Telemedicine in Cancer Care	50	9
4.	Using thematic analysis in psychology	50	2
5.	Consolidated criteria for reporting qualitative research (COREQ): a 32-item checklist for interviews and focus groups	47	14
6.	The Law of Attrition	47	7
7.	Cancer patients in SARS-CoV-2 infection: a nationwide analysis in China	46	5
8.	What is e-health?	44	10
9.	Recommendations on digital interventions for health system strengthening	43	6
10.	Social foundations of thought and action: A social cognitive theory.	43	3

Pre-2020, co-citation patterns were dominated by foundational digital health frameworks. Post-2020, a surge in citations related to COVID-19, AI-based interventions, and patient self-management tools reflects an evolving research focus.

### 5.11 Analysis of Social Knowledge Structure

#### 5.11.1 Authors' collaboration network analysis

**Figure 13:**
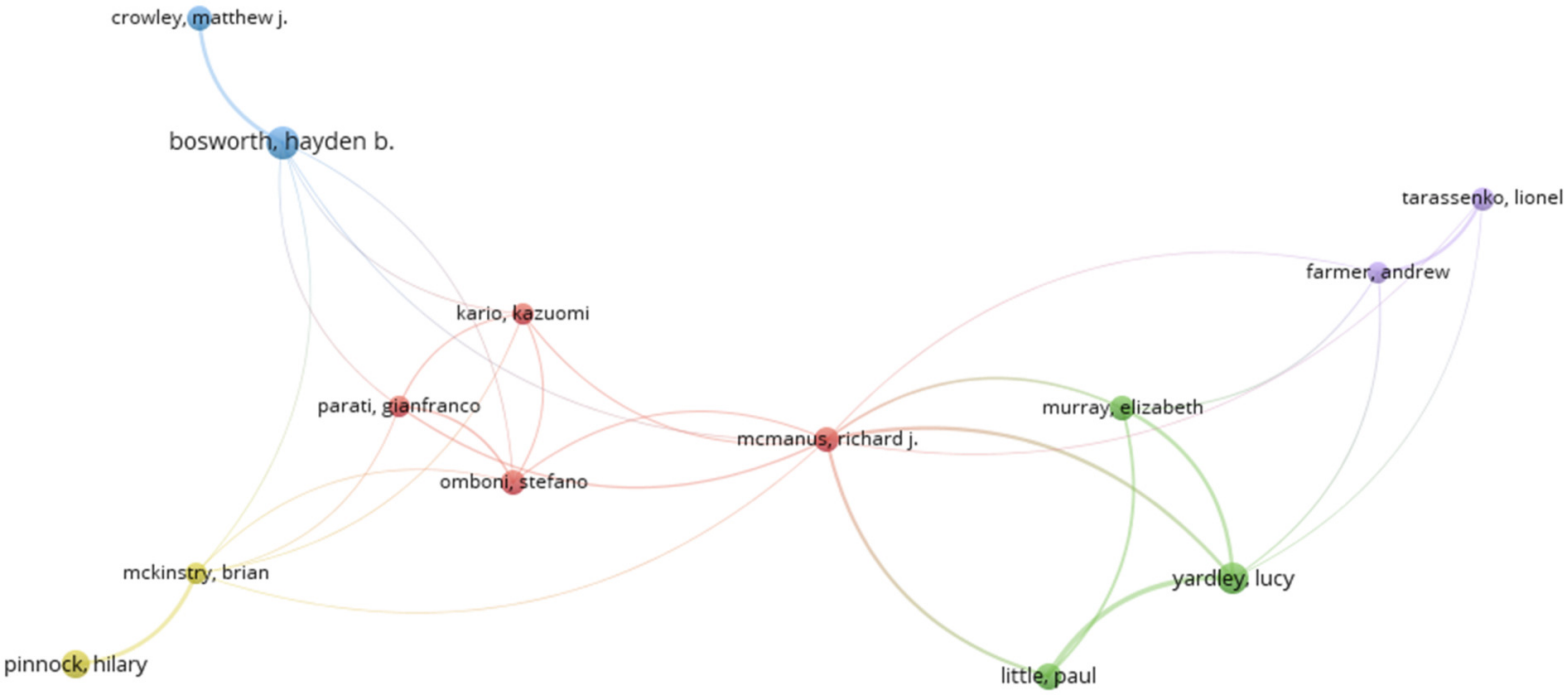
The network visualisation of co-authorship between the authors.

VOS viewer software was employed to map and visualise the relationships between authors based on their co-authored publications. This type of analysis helps researchers understand patterns of academic collaboration, such as which authors frequently work together and how these collaborations form larger clusters of research groups. Each node in the network represents an author. The lines connecting the nodes represent co-authorship relationships, where two authors have worked on one or more publications together. Authors who frequently collaborate with each other are grouped into clusters, which are typically represented by different colours. These clusters reflect collaborative research teams or communities. The size of each node indicates the author's productivity, often based on the number of publications. The thickness of the lines (edges) between authors can indicate the strength of collaboration, i.e., how many times two authors have co-published (53). This type of analysis is valuable for identifying influential researchers, key collaboration networks, and potential gaps or opportunities for new collaborations in a specific field of study. Choosing 15 as a minimum number of documents, out of 51,858 authors, 21 met the threshold highlights the co-authorship between them (Figure 13).

#### 5.11.2 Institution collaboration network analysis

**Figure 14:**
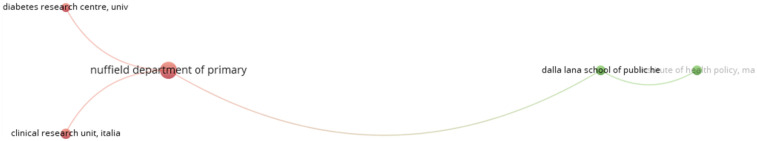
The network visualisation of co-authorship between the institutions.

VOS viewer software was employed to visualise co-authorship between institutions, helping to identify collaborative networks and track their evolution over time. The analysis focused on institutions that produced ten or more papers, with the results presented in Figure 14. In this visualisation, node size represented publication volume, links depicted co-authorship connections, and node colours indicated distinct clusters. From the 37,779 organisations, a minimum number of documents were chosen for 12 institutions that met the threshold and were included in the analysis. Instead of analysing individual authors, this approach aggregates data at the institutional level, focusing on how organisations collaborate with one another based on their authors' co-authored papers. The network shows organisations as nodes, with the connections (or edges) representing co-authorship ties. The more papers institutions co-author, the stronger the link between them in the network (54). The findings suggest that inter-institutional collaborations predominantly occur within national borders. Institutions with higher publication outputs tended to collaborate more frequently with others, indicating that fostering institutional partnerships could enhance the quality and quantity of research outputs.

#### 5.11.3 Countries collaboration network analysis

**Figure 15:**
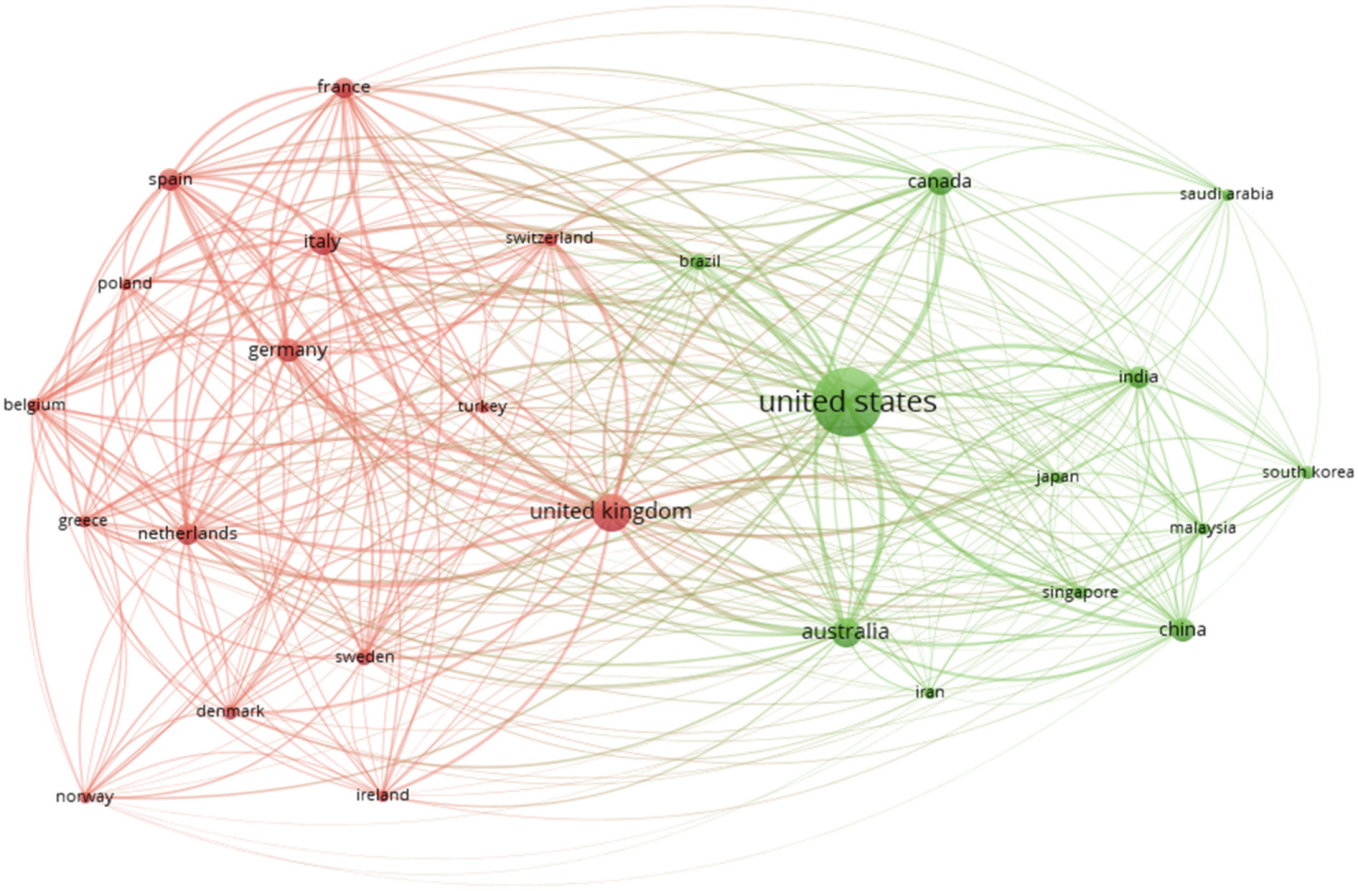
Snapshot of the bibliometric map representing Co-authorship analysis of countries in network visualisation mode.

Visualisation of collaboration among countries with a minimum productivity of 100 documents is shown in Figure 15 the analysis shows how authors from different countries collaborate. Each country is represented as a node, and the node size indicates the number of co-authored papers or the contribution level. The links between nodes (countries) represent collaborative relationships, with thicker links showing stronger or more frequent collaborations. The map showed 27 countries in three different clusters, each with a different colour (55).

The analysis highlights that the USA has the strongest research collaborations with other countries (link strength = 1,611), the UK (link strength = 1,606), Italy (link strength = 951), Germany (link strength = 840), Australia (Link Strength = 808), Spain (Link Strength = 761). The thickness of the connecting line between any two countries indicates the strength of collaboration. Countries with similar colours form one cluster.

## 6 Discussion

This study aimed to conduct a comprehensive bibliometric analysis of research on *digital interventions* for individuals living with noncommunicable diseases. The study finding reveals a substantial growth trend in *digital interventions* and NCDs research since 2021. Between 2018 and 2019, an exponential increase marked a period of rapid development, particularly noteworthy as annual publications consistently increased, peaking at 1,536 in 2021. The slight decrease in publications between 2021 and 2024 may suggest that while interest in this field remains strong, it may have stabilized after a period of rapid expansion, likely influenced by urgent research needs in the wake of COVID-19 and the ongoing demand for digital healthcare solutions.

Other factors included redirected focus from digital health for NCDs to emerging areas such as mental health, long COVID, and vaccination strategies (56–58). Changes in funding distribution post-pandemic could have led to reduced financial support for specific research domains (59). Additionally, delays in journal peer-review processes and publication backlogs may have slowed research dissemination. Technological advancements and evolving policy priorities might have influenced the diversification of research topics, leading to fewer publications in certain areas.

Emnet Getachew et al. highlighted the vital role of digital health technologies during the COVID-19 pandemic. These technologies contributed to tracking the spread of COVID-19, diagnosing patients, accelerating the search for treatments and vaccines, and supporting environmental disinfection efforts. Tools such as electronic health records, computerized clinical decision-making systems, telemedicine, and mobile health applications have demonstrated a strong potential to reinforce healthcare systems. Recently, digital health interventions have supported various aspects of the health sector, including prevention, early diagnosis, treatment adherence, medication safety, care coordination, documentation, data management, outbreak monitoring, and pandemic tracking (60–62).

The distribution of research across subject areas shows a dominant focus in Medicine, accounting for the majority, followed by Biochemistry, Genetics and Molecular Biology. Health Professions, Nursing, and Engineering also contribute significantly, along with Computer Science and Social Sciences. This distribution highlights that digital interventions and NCDs research is highly interdisciplinary, emphasizing the collaboration between clinical, technical, and social research sectors. Medicine's predominance underscores its central role in applying digital interventions to manage and prevent NCDs. A document published by WHO “Recommendations on digital interventions for health system strengthening” highlights the role of digital health interventions across medical, technical, and social domains in improving health outcomes and thus supports the findings that it is an inter-disciplinary topic (5). The field also attracts considerable authorship, with a total of 51,858 authors, ten of whom are particularly prolific, contributing to 2.5% of the total papers. Leading contributors include Bosworth, H.B. from the Duke Clinical Research Institute, Durham, and Yardley, L. from the UK Health Security Agency, London, who have achieved high citation counts, reflecting significant influence in this area focussing on Medication Compliance, Drug Therapy, Randomized Controlled Trial in digital interventions and NCDs. The most-cited work, “Psychosocial Impact of COVID-19” by Dubey et al., underscores the intersection of mental health and digital interventions research during the pandemic (37). Similarly, another influential article published by Hamine, Saee et al. and Marcolino, Milena Soriano et al. highlights the importance of the impact of mHealth interventions for chronic diseases (42, 44).

Institutionally, contributions are concentrated in leading universities and research institutions. Harvard Medical School ranks highest in publication output, followed by the University of Toronto and the University of California, San Francisco. Other notable institutions include the University of Sydney, Duke University School of Medicine, and Imperial College London. These top institutions contributed around 16.6% of total publications, emphasizing their critical role in advancing the field. Research funding further supports this trend, with the National Institutes of Health and the U.S. Department of Health and Human Services providing substantial backing, along with contributions from European, Australian, and UK institutions. This funding distribution underscores the USA's significant investment in digital health research, followed by contributions from the UK, Australia, and European countries.

The research themes identified through author keywords include “telemedicine”, “COVID-19”, “tele-health”, “digital health”, and “cancer”, which have seen notable increases since 2014, reflecting shifting research priorities over time. The popularity of these terms indicates a strong focus on digital tools and strategies to support self-management, patient engagement, and health outcomes for NCDs patients. A study conducted by Maria Armaou et al. highlighted that the rise in digital health interventions research since 2014 has been driven by technological advances, public health needs, and social demand for accessible healthcare. Key factors include the increased availability of mobile devices, wearable health technology, and internet access, which has expanded remote health service options, particularly in underserved communities. Moreover, the COVID-19 pandemic emphasized the importance of digital tools in mental health, chronic disease management, and preventative care. Studies show substantial growth in areas like mental well-being, tele-health, and chronic condition management, reflecting the field's adaptive response to modern health challenges (5, 63). Emerging terms after 2019, such as “conversational agents”, “colorectal cancer screening”, “artificial intelligence”, and “implementation science”, suggest an expanding interest in advanced technologies and specialized applications. This evolution is likely tied to the on-going need for efficient digital interventions to manage rising NCDs rates and healthcare demands, with digital health tools playing an increasingly prominent role.A study conducted by Hang Qiu et al. highlights ground-breaking research in machine learning; artificial intelligence (AI) has shown great application potential in diagnosing colorectal cancer (64). A study conducted by John D. McGreevey III, MD et al. in the year 2020 highlights the Clinical, Legal, and Ethical Aspects of Artificial Intelligence–Assisted Conversational Agents in Health Care (65).

Further thematic analysis of keywords provides insights into the maturity and relevance of different research areas. Themes like digital health, mHealth, eHealth, oncology, and cancer, classified as “motor themes”, indicate well-developed, high-impact areas essential to structuring the research field. Niche themes, such as melanoma and skin cancer, are mature but more specialized. A study conducted by Ravi B Parikh et al. highlights the importance of digital health applications in Oncology. The study explains that digital health innovation is relatively nascent in cancer care, and it is an opportunity to seize (66). A similar study conducted by Smit Patel et al. highlights the advancing digital health Innovation in Oncology, which should be Prioritised for High-Value digital Transformation in Cancer Care (67). Foundational themes like diabetes and hypertension require further development while emerging topics such as artificial intelligence and diabetic retinopathy are less developed but may gain future prominence as the field evolves. A study conducted by Jie Yao et al. in the year 2024 supports the explanation that there are challenges related to deployment, regulatory compliance, and patient privacy, which is essential for these technologies to realize their full potential (68). These findings demonstrate that while certain topics are well-established, others continue to develop or emerge as researchers adapt to evolving healthcare challenges. In analysing co-citation patterns, the study identified leading authors frequently referenced together, revealing significant research themes and networks within digital interventions and NCDs research. Highly cited authors like Wang Y. and Michie S. are influential, with the top ten authors contributing over 5,600 co-citations. Co-cited journals analysis identified a small group of journals central to the field, led by the *Journal of Medical Internet Research*, *Diabetes Care, JAMA*, and *The Lancet,* indicating their essential role in advancing digital ss research. This core group of journals aligns with Bradford's Law, which suggests that a limited number of journals in a field contain a substantial portion of relevant literature (69).

Co-authorship analysis reveals that collaborations are largely institution-based, with higher-output institutions demonstrating frequent partnerships. Institutions with larger publication volumes collaborate extensively within national borders, reflecting a trend toward intra-national research alliances. The institutes located in well-developed countries formed one cluster, whereas the countries located nearby formed one cluster. In a similar vein, international collaboration analysis shows that countries like the USA and the UK maintain strong research partnerships. High link strengths between these countries, and with others such as Italy, Germany, and Australia, reflect robust collaborative networks in digital health and NCDs research, fostering knowledge exchange and joint efforts in tackling global healthcare challenges.

With innovations like India's *m-Mitra* for maternal health, Kenya's *AfyaPap* for diabetes management, and Brazil's *Telehealth Network*, LMICs leverage mobile health and telemedicine to combat NCDs. However, they face significant barriers, including **funding deficits** (limited research grants), **infrastructure gaps** (poor internet connectivity in rural areas), **regulatory hurdles** (lack of standardized digital health policies), **health system strain** (shortage of trained healthcare professionals), and **low literacy** (limited digital and health awareness among populations).

Overall, the field of *digital interventions* for NCDs is marked by active, cross-disciplinary research with significant institutional and international collaboration. The support from leading institutions, researchers, and journals suggests a highly engaged academic community, and the ongoing focus on emerging technologies and digital tools is likely to shape future directions in healthcare innovation.

This bibliometric analysis provides valuable insights that can guide future research and policy development in digital interventions for NCDs. Policymakers can use these findings to identify research gaps, allocate funding strategically, and develop regulatory frameworks that support evidence-based digital health solutions. For instance, the observed research trends highlight the growing focus on telemedicine, AI-driven interventions, and patient self-management, suggesting priority areas for investment in healthcare innovation. For researchers, this study offers a comprehensive map of influential studies, emerging themes, and collaborative networks. By leveraging keyword co-occurrence and citation analysis, researchers can refine their focus on underexplored areas such as digital health applications in LMICs or real-world implementation of AI-driven NCDs management. Additionally, co-authorship and institutional collaboration patterns highlight potential partnerships that can foster multidisciplinary research. Integrating these findings into policy and research agendas can enhance digital health adoption, drive innovation, and improve NCDs management globally.

## 7 Limitations of this study

A limitation of this study is its reliance on publicly available databases, primarily Scopus, which may miss relevant publications, especially those in non-indexed journals, gray literature, or non-English sources. Additionally, the study focuses solely on the period between1 January 2004 and Sept 30, 2024, potentially overlooking emerging trends beyond 2024. It does not assess the quality or effectiveness of digital interventions for NCDs, nor does it explore regional differences in research output influenced by varying infrastructure and funding. Moreover, biases in citation practices or author self-citation may skew the analysis.

## 8 Conclusion

This bibliometric analysis highlights the critical role of digital interventions in advancing NCDs management by improving accessibility, patient self-management, and healthcare system efficiency. The findings underscore the increasing integration of telemedicine, AI-driven diagnostics, and mobile health applications, reflecting a shift toward technology-driven care models. Actionable insights from this study suggest that **policymakers** should prioritize investment in digital health infrastructure, develop regulatory frameworks to standardize digital interventions and ensure equitable access, particularly in LMICs. **Researchers** can leverage emerging trends, such as AI-enhanced predictive analytics and personalized digital therapeutics, to address existing gaps and enhance interventions effectiveness. Strengthening interdisciplinary collaborations and expanding research in underrepresented regions will be key to optimizing digital solutions for NCDs prevention and management. By aligning policy and research efforts with these evolving digital health trends, stakeholders can drive innovation and enhance healthcare outcomes for individuals living with NCDs.

## Data Availability

The original contributions presented in the study are included in the article/Supplementary Material, further inquiries can be directed to the corresponding author.
